# Association of current and former smoking with body mass index: A study of smoking discordant twin pairs from 21 twin cohorts

**DOI:** 10.1371/journal.pone.0200140

**Published:** 2018-07-12

**Authors:** Maarit Piirtola, Aline Jelenkovic, Antti Latvala, Reijo Sund, Chika Honda, Fujio Inui, Mikio Watanabe, Rie Tomizawa, Yoshinori Iwatani, Juan R. Ordoñana, Juan F. Sánchez-Romera, Lucia Colodro-Conde, Adam D. Tarnoki, David L. Tarnoki, Nicholas G. Martin, Grant W. Montgomery, Sarah E. Medland, Finn Rasmussen, Per Tynelius, Qihua Tan, Dongfeng Zhang, Zengchang Pang, Esther Rebato, Maria A. Stazi, Corrado Fagnani, Sonia Brescianini, Andreas Busjahn, Jennifer R. Harris, Ingunn Brandt, Thomas Sevenius Nilsen, Tessa L. Cutler, John L. Hopper, Robin P. Corley, Brooke M. Huibregtse, Joohon Sung, Jina Kim, Jooyeon Lee, Sooji Lee, Margaret Gatz, David A. Butler, Carol E. Franz, William S. Kremen, Michael J. Lyons, Patrik K. E. Magnusson, Nancy L. Pedersen, Anna K. Dahl Aslan, Sevgi Y. Öncel, Fazil Aliev, Catherine A. Derom, Robert F. Vlietinck, Ruth J. F. Loos, Judy L. Silberg, Hermine H. Maes, Dorret I. Boomsma, Thorkild I. A. Sørensen, Tellervo Korhonen, Jaakko Kaprio, Karri Silventoinen

**Affiliations:** 1 Department of Social Research, University of Helsinki, Helsinki, Finland; 2 Institute for Molecular Medicine Finland (FIMM), University of Helsinki, Helsinki, Finland; 3 Department of Genetics, Physical Anthropology and Animal Physiology, University of the Basque Country UPV/EHU, Leioa, Spain; 4 Department of Public Health, University of Helsinki, Helsinki, Finland; 5 Institute of Clinical Medicine, University of Eastern Finland, Kuopio, Finland; 6 Osaka University Graduate School of Medicine, Osaka University, Osaka, Japan; 7 Faculty of Health Science, Kio University, Nara, Japan; 8 Department of Human Anatomy and Psychobiology, University of Murcia, Murcia, Spain; 9 IMIB-Arrixaca, Murcia, Spain; 10 Department of Developmental and Educational Psychology, University of Murcia, Murcia, Spain; 11 QIMR Berghofer Medical Research Institute, Brisbane, Australia; 12 Department of Radiology, Semmelweis University, Budapest, Hungary; 13 Hungarian Twin Registry, Budapest, Hungary; 14 Department of Public Health Sciences, Karolinska Institutet, Stockholm, Sweden; 15 Epidemiology, Biostatistics and Biodemography, Department of Public Health, University of Southern Denmark, Odense, Denmark; 16 Department of Public Health, Qingdao University Medical College, Qingdao, China; 17 Department of Noncommunicable Diseases Prevention, Qingdao Centers for Disease Control and Prevention, Qingdao, China; 18 Istituto Superiore di Sanità—Centre for Behavioural Sciences and Mental Health, Rome, Italy; 19 HealthTwiSt GmbH, Berlin, Germany; 20 Norwegian Institute of Public Health, Oslo, Norway; 21 Twins Research Australia, Centre for Epidemiology and Biostatistics, The University of Melbourne, Melbourne, Victoria, Australia; 22 Department of Epidemiology, School of Public Health, Seoul National University, Seoul, South Korea; 23 Institute for Behavioral Genetics, University of Colorado, Boulder, CO, United States of America; 24 Institute of Health and Environment, Seoul National University, Seoul, South Korea; 25 Center for Economic and Social Research, University of Southern California, Los Angeles, CA, United States of America; 26 Department of Medical Epidemiology and Biostatistics, Karolinska Institutet, Stockholm, Sweden; 27 Health and Medicine Division, The National Academies of Sciences, Engineering, and Medicine, Washington, DC, United States of America; 28 Department of Psychiatry, University of California, San Diego, CA, United States of America; 29 VA San Diego Center of Excellence for Stress and Mental Health, La Jolla, CA, United States of America; 30 Department of Psychology, Boston University, Boston, MA, United States of America; 31 Institute of Gerontology and Aging Research Network–Jönköping (ARN-J), School of Health and Welfare, Jönköping University, Jönköping, Sweden; 32 Department of Statistics, Faculty of Arts and Sciences, Kırıkkale University, Kırıkkale, Turkey; 33 Psychology and African American Studies, Virginia Commonwealth University, Richmond, VA, United States of America; 34 Faculty of Business, Karabuk University, Karabuk, Turkey; 35 Centre of Human Genetics, University Hospitals Leuven, Leuven, Belgium; 36 Department of Obstetrics and Gynaecology, Ghent University Hospitals, Ghent, Belgium; 37 The Charles Bronfman Institute for Personalized Medicine, The Mindich Child Health and Development Institute, Icahn School of Medicine at Mount Sinai, New York, NY, United States of America; 38 Department of Human and Molecular Genetics, Virginia Institute for Psychiatric and Behavioral Genetics, Virginia Commonwealth University, Richmond, VA, United States of America; 39 Department of Human and Molecular Genetics, Psychiatry & Massey Cancer Center, Virginia Commonwealth University, Richmond, VA, United States of America; 40 Department of Biological Psychology, VU University Amsterdam, Amsterdam, Netherlands; 41 Novo Nordisk Foundation Centre for Basic Metabolic Research (Section for Metabolic Genetics), Faculty of Health and Medical Sciences, University of Copenhagen, Copenhagen, Denmark; 42 Department of Public Health (Section of Epidemiology), Faculty of Health and Medical Sciences, University of Copenhagen, Copenhagen, Denmark; Istituto Di Ricerche Farmacologiche Mario Negri, ITALY

## Abstract

**Background:**

Smokers tend to weigh less than never smokers, while successful quitting leads to an increase in body weight. Because smokers and non-smokers may differ in genetic and environmental family background, we analysed data from twin pairs in which the co-twins differed by their smoking behaviour to evaluate if the association between smoking and body mass index (BMI) remains after controlling for family background.

**Methods and findings:**

The international CODATwins database includes information on smoking and BMI measured between 1960 and 2012 from 156,593 twin individuals 18–69 years of age. Individual-based data (230,378 measurements) and data of smoking discordant twin pairs (altogether 30,014 pairwise measurements, 36% from monozygotic [MZ] pairs) were analysed with linear fixed-effects regression models by 10-year periods. In MZ pairs, the smoking co-twin had, on average, 0.57 kg/m^2^ lower BMI in men (95% confidence interval (CI): 0.49, 0.70) and 0.65 kg/m^2^ lower BMI in women (95% CI: 0.52, 0.79) than the never smoking co-twin. Former smokers had 0.70 kg/m^2^ higher BMI among men (95% CI: 0.63, 0.78) and 0.62 kg/m^2^ higher BMI among women (95% CI: 0.51, 0.73) than their currently smoking MZ co-twins. Little difference in BMI was observed when comparing former smoking co-twins with their never smoking MZ co-twins (0.13 kg/m^2^, 95% CI 0.04, 0.23 among men; -0.04 kg/m^2^, 95% CI -0.16, 0.09 among women). The associations were similar within dizygotic pairs and when analysing twins as individuals. The observed series of cross-sectional associations were independent of sex, age, and measurement decade.

**Conclusions:**

Smoking is associated with lower BMI and smoking cessation with higher BMI. However, the net effect of smoking and subsequent cessation on weight development appears to be minimal, i.e. never more than an average of 0.7 kg/m^2^.

## Introduction

Smoking and obesity are among the leading modifiable risk factors for many non-communicable diseases, contributing to an increased risk of premature death and rising healthcare costs [1, 2]. While smoking prevalence has globally decreased during the last decades, especially in high-income countries, body mass index (BMI, kg/m^2^) has increased during the same time period [2, 3]. There is a common belief that smoking controls weight and that quitting leads to increases in body weight [4, 5]. The causal association of smoking and changes in smoking with BMI is, however, unclear.

Smoking and nicotine are suggested to reduce weight both by increasing energy expenditure and by suppressing appetite [4]. On average, current smokers have lower BMIs than never smokers [6–11]. This association has been systematic in large population-based cohorts in both cross-sectional and longitudinal designs, and even in Mendelian randomisation (MR) meta-analyses testing the molecular mechanisms and causality behind smoking and BMI [6–11]. The causal effects of nicotine and other components of tobacco smoke on BMI are also supported by evidence that those who successfully quit smoking tend to gain, on average, 0.63 kg/m^2^ in BMI, compared to those who continue to smoke [5].

There is, however, also evidence against causality between smoking and BMI. First, not all quitters gain weight after smoking cessation [12]. Second, smoking quantity does not have a linear dose–response association with weight: heavy smokers have had higher BMI and higher central adiposity than light smokers, even after controlling for other lifestyle factors and socio-demographic background [8, 10, 13]. Third, both smoking [14] and BMI [15] have moderate-to-strong underlying genetic components [16], and specific genetic variants have been identified to be associated with BMI and smoking explaining potentially the association [17]. It has also been shown that smoking has effects on DNA methylation and on gene expression which are potentially reversible [18, 19]. Complexity related to the effects of smoking on BMI has been evident in MR studies, in which the same genetic variant allele was associated with lower BMI in current smokers but with higher BMI in never smokers [20]. This finding suggests that genetic variants influence BMI, via smoking, other behavioural factors and environmental confounders. The importance of controlling for genetic factors underlying the association between smoking and BMI has also been suggested by a genome-wide meta-analysis [21]. Notably, MR and twin designs are based on totally different principles and assumptions [22, 23].

In summary, those who initiate smoking might differ from non-smokers, not only in their BMI and health-related behaviours before smoking initiation, but also in their genotype and many environmental exposures [24]. Furthermore, quitters differ in many ways from those who continue smoking; with respect to, for example, education level, employment status, health behaviours and other psychosocial factors [25]. Therefore, determining causation between quitting smoking and weight gain is not straightforward and a design that includes twin pairs, who share not only genes but generally also much of their early life exposures and experiences, can shed more light on the causal associations between smoking and BMI.

Our aim was to test the hypothesis of association between smoking and BMI in a discordant twin design. In particular, we focused on monozygotic (MZ, i.e. genetically identical) twin pairs who differ for smoking status. To confirm the consistency of associations, we analysed the data separately in men and women, as well as by zygosity and decade of data collection (from the 1960s to 2012).

## Methods

### Study design, participants and measures

The data were derived from the CODATwins (COllaborative project of Development of Anthropometrical measures in Twins) database. The CODATwins project aimed to pool all existing twin data on height and weight in the world, as previously described in detail [26], and has been carried out according to the ethical principles expressed in the Declaration of Helsinki. All participants were volunteers who gave informed consent when participating in their original studies. Only a limited set of observational variables and anonymised data were delivered to the data-management center at the University of Helsinki. The pooled analysis was approved by the ethical committee of the Department of Public Health, University of Helsinki, and the methods were carried out in accordance with the approved guidelines.

**Fig 1:**
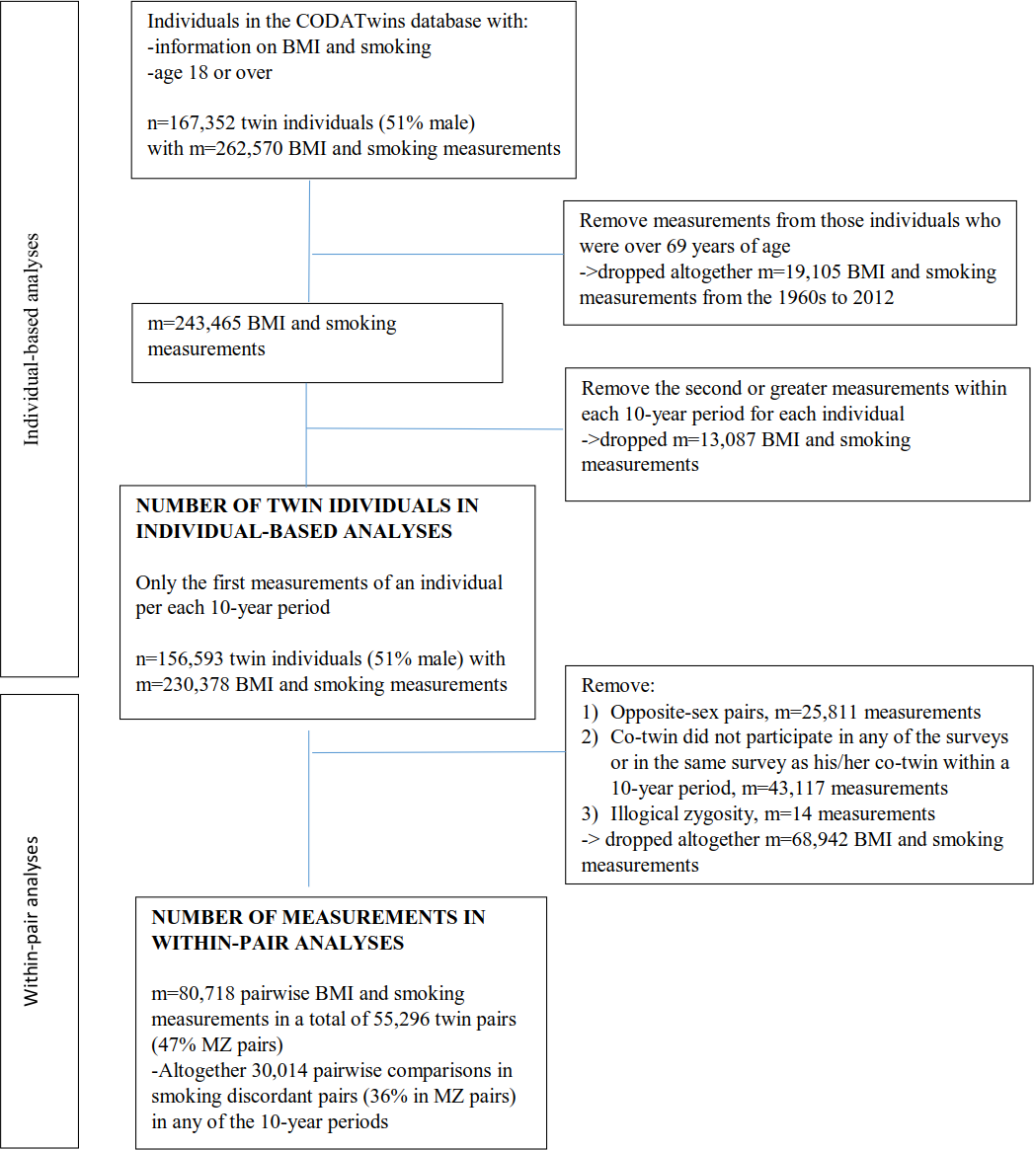
Flow chart of the CODATwins dataset (n = 156,593 twin individuals and 30,014 pairwise comparisons in smoking discordant same-sexed twin pairs) included in the study. BMI = body mass index; MZ = monozygotic.

From the database, we selected twins aged 18 through 69 years at the time of measurements with information on both BMI and smoking status (Fig 1). This provided 156,593 individuals (51% men), with a total of 230,378 BMI and smoking measurements (mean age at measurement of 41.9 [standard deviation (SD) 14.1] years) from 21 twin cohorts representing 14 countries (Fig 1, S1 Table). Of all individuals, we included 55,296 same-sexed twin pairs (47% MZ pairs) for pairwise analyses. From the pairs, 35,909 pairs had one, 14,772 pairs had two, 3,195 pairs three and 1,420 pairs four pairwise measurements between 1960 and 2012 in the dataset (Fig 1). The majority (97%) of weight and height measures used for calculating BMI (kg/m^2^) were self-reported values. Smoking status was categorised as never smokers, current smokers (daily and occasional) and former smokers (i.e., those who had smoked occasionally or regularly in the past, but did not smoke at the time of data collection). Occasional smokers were separately identified in only three cohorts. For those cohorts, we decided to pool occasional smokers and current smokers together in order to maintain a pure reference group of never smokers, and to consider any exposure to smoking as a sufficient criterion for being a current smoker.

### Analytical strategy

There are two kinds of twins: dizygotic (DZ, i.e. fraternal) twins who share, on average, 50% of genes identical-by-descent and MZ twins who share virtually 100% of their genomic sequences. In particular, smoking discordant MZ pairs allow for controlling for sex, birth cohort, and genetic factors, as well as for many of the environmental experiences and exposures [23]. DZ twins share many of the demographic and environmental exposures but have different genotypes. By comparing BMI in smoking twins to BMI of non-smoking co-twins as a function of zygosity and by comparing these associations to the association in smokers and non-smokers as individuals, it is possible to gain insight into the causal effect of smoking on BMI [27].

First, we performed individual-based analyses (i.e., twins within a pair and single co-twins were studied as individuals) to evaluate if the epidemiological association between smoking behaviour and BMI seen in other population-based studies is also present in the twin data.

The analyses within twin pairs, discordant for their smoking status, provide information regarding the role of genetic and shared environmental familial factors in the association between smoking and BMI [27, 28]. Notably, results from within-pair analyses should be interpreted by comparing them with individual-based results. This design has been previously described in more detail [23, 27, 29]. Briefly, unmeasured familial confounders which cannot be taken into account in individual analyses are controlled for in within-pair analyses, which by design rule out all factors shared by co-twins. If confounding by the shared environment plays a role in the association between smoking and BMI, the association observed among all individuals would be attenuated within both DZ and MZ twin pairs discordant for their smoking status. In the case of solely genetic confounding, the association would be present among individuals, attenuated within DZ pairs and reduced or non-existent within MZ pairs, where all genetic differences are ruled out. In contrast, similar associations at the individual level and within both DZ and MZ pairs would indicate that the association between smoking and BMI is independent of genetic and shared environmental familial factors. Thus, individual-specific environmental factors (such as smoking by only one co-twin) would result in differences within MZ pairs. In the case of a causal model, all within-pair differences in smoking will result in within-pair differences in BMI [27].

### Statistical analyses

In *the individual-based analyses*, the association of being a smoker with BMI was analysed by comparing current smokers with never smokers used as the reference category. The association between smoking cessation and BMI was analysed by comparing those who quit smoking (former smokers) with the current smokers (reference), and finally the net effect of smoking cessation on BMI was analysed by comparing former smokers to never smokers (reference). Based on previous findings, we proposed a hypothesis that smoking might be associated with BMI differently by sex and time-periods [9, 30, 31]. Since also a likelihood ratio test showed statistically significant interactions on BMI between smoking status and sex and smoking status and 10-year measurement time periods (both p-values <0.001), data from men and women were analysed separately by 10-year measurement periods. Only one measurement per individual per 10-year period was allowed. In the case of multiple observations during a 10-year period, the earliest measurement for an individual was selected within each 10-year period (Fig 1). Linear regression analyses were used to analyse the association between smoking status and BMI pooled over time and by each 10-year period. To adjust for the non-independence of observations within twin pairs, an estimator was used to take into account clustering by twin pair identifier [32]. All analyses were adjusted for age, age squared (to take into account the nonlinearity of age distributions in the data) and twin cohort (i.e., different twin databases which might come from different countries).

Then, we performed *within-pair analyses* in twin pairs discordant for their smoking status. These analyses were restricted to same-sex twin pairs with non-missing data for both twins within a pair during each 10-year period (Fig 1). Analyses were performed in the same order as in the individual-based analyses. First, pairs in which one twin was a current smoker and the co-twin had never smoked were used to demonstrate the effect of becoming a smoker on BMI, independent of genetic and shared environmental familial factors. Second, we compared BMI in pairs in which one twin was a current smoker and the co-twin had quit smoking to demonstrate the effects of cessation on BMI. Third, to study the net effect of smoking cessation, we compared the pairs in which one twin had never smoked and his/her co-twin was a former smoker. However, because we allowed twin pairs to contribute data within each 10-year period, it was possible that measurements for co-twins were performed at different times. Therefore, age and age squared differences between pairs were also adjusted for in the within-pair analyses. Within-pair analyses were performed using linear fixed-effects regression models separately by sex, zygosity and 10-year period [33]. Stata SE version 14.1 (StataCorp, College Station, Texas, USA) was used for all the analyses.

The heterogeneity of the mean changes in the magnitude of BMI estimates for the three smoking behaviour comparisons over time (i.e., variation in BMI estimates attributable to heterogeneity between 10-year time periods) were analysed by using I-squared tests separately by sex and zygosity [34]. Summary statistics were used in the meta-analysis in which the dependence of using twins has already been taken into account in generating the standard errors. Heterogeneity analyses were conducted with the metan-procedure in Stata.

## Results

The distributions of smoking status and BMI by sex and 10-year periods are described in Table 1. In both sexes and in all smoking categories, the mean BMI values were highest after 1999. Detailed BMI values by smoking categories and twin cohorts are shown in S1 Table.

**Table 1:** Descriptive statistics of age and BMI (kg/m^2^) by smoking status over time between 1960 and 2012 in 156,593 twin individuals (80,384 men; 76,210 women) with 30,014 smoking discordant pairwise measurements in the CODATwins database.

Time period	Number of BMI/ smoking observations	Age mean (SD)	BMI by smoking status						Number of smoking discordant pairs and/or pairwise comparisons
			Never		Current		Former		
			n (%)	mean (SD)	n (%)	mean (SD)	n (%)	mean (SD)	
**Men**									
1960–69	10,460 ^b^	44.1 (2.9)	2,806 (27)	25.2 (2.7)	4,996 (48)	24.5 (2.8)	2,658 (25)	25.2 (2.6)	1,792 ^c^
1970–79	27,168 ^b^	34.8 (11.7)	10,014 (37)	23.4 (2.9)	10,756 (40)	23.4 (2.9)	6,398 (24)	24.2 (3.0)	4,740 ^c^
1980–89	30,338 ^b^	45.2 (15.4)	10,401 (34)	24.3 (3.0)	9,686 (32)	24.0 (3.1)	10,251 (34)	25.3 (3.1)	4,274 ^c^
1990–99	22,348 ^b^	46.1 (14.3)	9,918 (44)	24.6 (3.2)	5,877 (26)	24.8 (3.3)	6,553 (29)	25.9 (3.2)	2,301 ^c^
2000–12	28,419 ^b^	44.0 (14.1)	13,475 (47)	25.2 (3.5)	7,381 (26)	25.0 (3.7)	7,563 (27)	26.5 (3.8)	3,204 ^c^
1960–2012	118,733 ^a^	42.6 (14.0)	46,614 (39)	24.5 (3.2)	38,696 (33)	24.2 (3.2)	33,423 (28)	25.5 (3.3)	16,311 ^d^
**Women**									
1970–79	26,604 ^b^	33.6 (11.5)	14,945 (56)	22.5 (3.4)	8,484 (32)	21.2 (2.8)	3,175 (12)	21.8 (3.0)	3,957 ^c^
1980–89	27,829 ^b^	39.4 (13.8)	15,046 (54)	23.2 (3.8)	7,750 (28)	22.0 (3.3)	5,033 (18)	22.7 (3.7)	3,798 ^c^
1990–99	24,004 ^b^	48.3 (12.9)	13,286 (55)	24.3 (3.9)	5,565 (23)	23.4 (3.7)	5,153 (21)	24.3 (3.9)	2,515 ^c^
2000–12	33,207 ^b^	43.8 (13.9)	18,865 (57)	23.9 (4.3)	7,559 (23)	23.7 (4.2)	6,784 (20)	24.6 (4.2)	3,433 ^c^
1960–2012	111,645 ^a^	41.2 (14.1)	62,142 (56)	23.5 (4.0)	29,358 (26)	22.5 (3.7)	20,145 (18)	23.6 (4.0)	13,703 ^d^

^a^ Total number of BMI/smoking measurements from 1960–2012. Some individuals were included multiple times in the data (i.e., in several 10-year periods).

^b^ Only one smoking status and BMI measurement for each individual per each 10-year time period.

^c^ Number of twin pairs (both dizygotic and monozygotic pairs) discordant for their smoking status per a 10-year time period. A pair could be included only once for each 10-year period.

^d^ Total number of smoking discordant pairwise measurements for 1960–2012. Note, each twin pair could be either concordant for smoking (i.e., same smoking status within a pair) or discordant for smoking (status differed within a pair: current-never, former-never, former-current) during each 10-year period. This number includes all discordant pairwise measurements/comparisons during 1960–2012.

BMI = body mass index; SD = standard deviation

### Individual level associations

**Fig 2:**
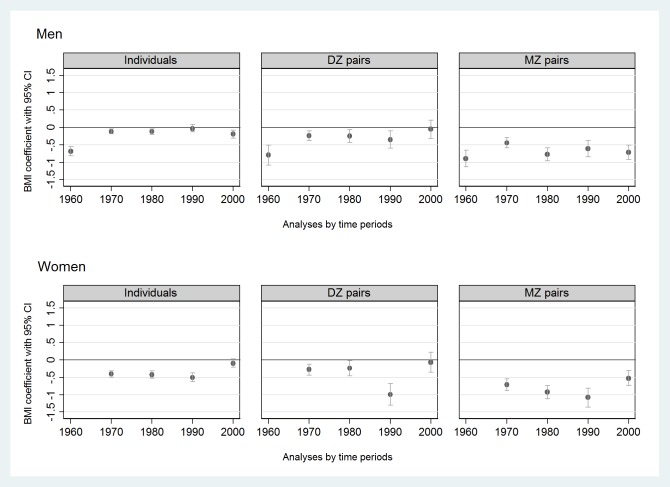
Associations (expressed by regression coefficients with 95% CIs, BMI units (kg/m^2^)) of current smoking with BMI compared to never smokers (reference) in twin individuals (n = 156,593) and same-sex twin pairs (DZ or MZ pairs) discordant for their smoking status (m = 10,128 pairwise measurements) by sex and time period from the CODATwins database, 1960–2012. BMI = body mass index; CI = confidence interval; DZ = dizygotic; MZ = monozygotic.

In the individual level data pooled over time (S2 Table, left column), current smokers had lower BMIs in both sexes than never smokers (β = -0.19 kg/m^2^ [95% CI -0.25, -0.14] in men and β = -0.35 kg/m^2^ [95% CI -0.41, -0.28] in women). There was high heterogeneity in BMI estimates (I^2^ was 94% in men and 88% in women) between data collection time periods, but no clear trend in time was seen (Fig 2).

**Fig 3:**
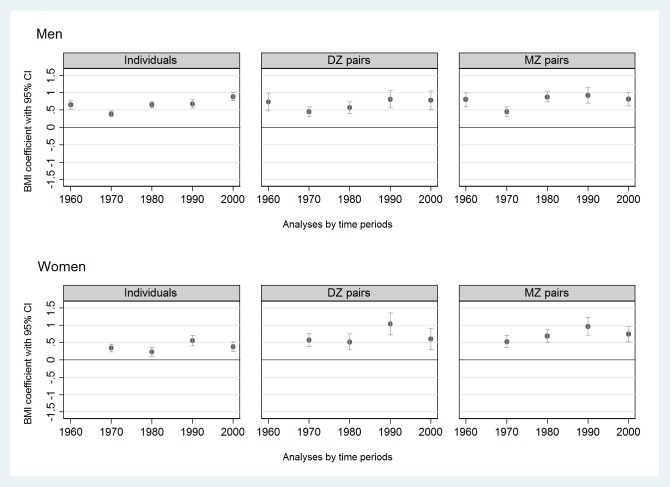
Associations (expressed by regression coefficients with 95% CIs, BMI units (kg/m^2^)) of former smoking with BMI compared to current smokers (reference) in twin individuals (n = 156,593) and same-sex twin pairs (DZ or MZ pairs) discordant for their smoking status (m = 10,551 pairwise measurements) by sex and time period from the CODATwins database, 1960–2012. BMI = body mass index; CI = confidence interval; DZ = dizygotic; MZ = monozygotic.

When using current smokers as the reference group, former smokers had a higher BMI in both sexes over the decades (pooled β = 0.66 kg/m^2^ [95% CI 0.61, 0.72] in men; pooled β = 0.43 kg/m^2^ [95% CI 0.36, 0.50] in women) (Fig 3, S3 Table). High heterogeneity by time period was seen in both sexes (I^2^ was 92% in men and 73% in women) without a clear trend in time (Fig 3).

**Fig 4:**
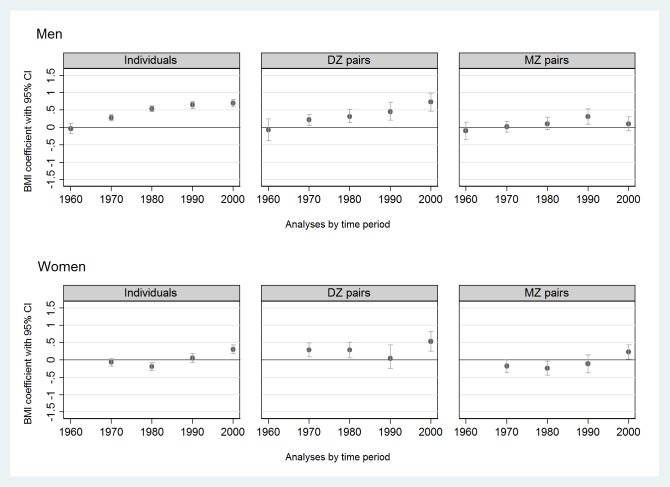
Associations (expressed by regression coefficients with 95% CIs, BMI units (kg/m^2^)) of former smoking with BMI compared to never smokers (reference) in twin individuals (n = 156,593) and same-sex twin pairs (DZ or MZ pairs) discordant for their smoking status (m = 9,336 pairwise measurements) by sex and time period from the CODATwins database, 1960–2012. BMI = body mass index; CI = confidence interval; DZ = dizygotic; MZ = monozygotic.

When comparing former smokers with never smokers, higher BMIs in men (pooled β over time = 0.46 kg/m^2^ [95% CI 0.41, 0.52]) and slightly higher BMIs in women (pooled β over time = 0.09 kg/m^2^ [95% CI 0.01, 0.16]) were found for former smokers (Fig 4, S4 Table). The magnitude of the associations fluctuated over time (I^2^ was 96% in men and 92% in women), and intrapair BMI estimates (i.e., BMI differences) were increasing from the 1960s among men and from the 1970s among women (Fig 4).

### Within-pair associations

Results from MZ and DZ pairs discordant for their smoking status are shown in Figs 2–4 (Fig 2, Fig 3, Fig 4) and in Supplement tables (S2–S4 Tables, last two columns). Compared to never smokers, current smokers had lower BMIs within both DZ and MZ pairs studied separately in all time periods in both sexes (S2 Table). The magnitude of the association within MZ pairs was approximately twice the magnitude of the association within DZ pairs (Fig 2). Former smokers had higher BMIs than current smokers in both DZ and MZ pairs and in both sexes (Fig 3, S3 Table). Finally, former smokers had higher BMIs than never smokers within DZ pairs but not within MZ pairs, exceptions being when a weak positive association was found among men in 1990–99 and among women in 2000–12 (Fig 4, S4 Table).

## Discussion

This study, with a series of cross-sectional analyses based on pooled data from 21 twin cohorts, confirmed and provided novel insights on the causal nature of the associations between smoking behaviour and BMI. The special novelty of the study is that it compares three types (current-never, former-current and former-never) of smoking discordant male and female MZ pairs in five different decades. In this study, current smokers had lower BMIs when compared with either never smokers or former smokers. When we examined the associations in MZ twin pairs, these associations remained significant, suggesting that smoking is associated with lower BMI, and quitting smoking is associated with greater BMI. However, comparing former smokers with never smokers, the net effect of smoking initiation followed by smoking cessation on BMI appears to be minimal when the effects of genetic and shared environmental family background are taken into account.

The combined implication of comparing current smokers, former smokers, and never smokers with this twin design is that even though quitting smoking may lead to higher BMI after smoking cessation, smoking does not affect the profile of weight development once genes and familial effects are accounted for. Our finding is supported by follow-up studies in which quitters’ BMIs were lower while they smoked but after quitting their BMIs increased to the same level of those who had never smoked [6, 9, 35, 36]. Notably, previous cohort studies and meta-analyses [5–9, 35, 36] were not able to adjust genetic and non-genetic familial background in their analyses as made possible by the twin study design in this study. By analysing multiple twin cohorts from different time periods, our study also contributes with the new information that despite the heterogeneity in average BMI over time, the results were generally consistent in both men and women and across all 10-year periods, especially in MZ pairs. These are important findings because of the current debate and widespread public perception about the effect of smoking on weight control in younger birth cohorts today [31].

Using twins as individuals, we showed that the associations of BMI and smoking in twins do not differ from those in the general population [5–9, 35, 36]. Our analyses provided similar associations between smoking and BMI that have been shown in other population-based studies: current smoking is associated with lower BMI and smoking cessation with higher BMI, compared with never smokers [5–9, 35]. A major strength of our study is that we were able to analyse data from MZ pairs discordant for their smoking status. Previously, there have only been few twin studies in MZ pairs, but they have been consistent with their conclusion that BMI is lower in smokers than in never smokers [37, 38]. Notably, those studies were based on male twins only and a majority of the twin individuals in those prior studies are not included in the CODATwins database [37, 38]. Our results, together with these earlier findings, suggest that smoking is associated with weight independently of genetic factors seen in both sexes. Importantly, our finding that the association between current smoking and lower BMI was consistently stronger within smoking discordant MZ pairs than smoking discordant DZ pairs suggests that smoking may be associated with lower BMI independently of genetic or shared environmental familial confounding.

In general, our findings support previous evidence that smoking cessation is associated with weight gain, seen in our data as higher BMI compared with those continuing smoking [35]. However, our results related to the BMI after smoking cessation compared with never smokers are worth highlighting. In previous studies, the BMI of former smokers has been higher than the BMI of never smokers [6, 7, 9, 35], an effect also evident in male MZ twin pairs [37, 38]. Based on our individual-level analyses, the evidence of higher BMI after smoking cessation was less clear in women even though more weight gain after smoking cessation has been reported, especially in women [9, 39]. Furthermore, when comparing the BMI of former smokers to the BMI of never smokers in within-pair analyses, the association disappeared or was attenuated, particularly in the MZ within-pair comparisons. Therefore, the effect of tobacco exposure on weight among persons who have initiated and then quit smoking seems to be nil or very small. Our finding is supported by two previous twin studies in which formerly smoking twins gained weight to approximately the level of their non-smoking co-twins [12, 40]. There is also previous evidence that after a decade post-cessation, former smokers’ BMIs do not differ substantially from those of never smokers [6, 9]. However, our analyses are cross-sectional in nature, therefore, we cannot exclude the possibility of reverse causality between BMI and smoking behaviour. Further, we do not have information on the pre-smoking initiation weight of current smokers nor information on weight immediately before a participant has quit smoking. A longitudinal study has indicated more weight gain after quitting smoking among former heavy smokers and among those already obese before quitting [41]. Notably, excess weight gainers have also been shown to differ in their health habits compared to modest weight gainers before quitting smoking [12]. Therefore, other factors than smoking quantity seem to control weight gain after smoking cessation. In our analyses, we could not evaluate the effect of smoking quantity or time since quitting. Increased eating (as a behaviour compensating for not having tobacco to smoke) is possible, but was not supported in either a population-based survey in which dietary energy density of former smokers was reported to be almost at the same level as that of non-smokers [42] or in men who quit smoking and whose calorie and alcohol consumption were followed for a 14-year period [6].

Our study has several strengths. We could rely on a unique database that covers 230,378 measurements of both smoking and BMI over a 50-year period in men and women. In addition to the extensive individual-based analyses, the twin design provided information on the independent effect of smoking on weight status by comparing a twin sister or brother who had never smoked to their co-twin who had initiated smoking and then quit. The power of within-pair analysis is that it controls for all unobserved factors constant within twin pairs (i.e., age, sex, cohort and all genetic and shared environmental familial factors shared by the co-twins) [23]. The within-pair analyses confirmed expected results for the independent associations of smoking initiation and cessation with BMI. These results extend previous evidence and give new evidence in that they also provide information for women, since previous studies regarding the effect of smoking initiation and cessation on BMI have provided information on male twin pairs only [12, 37, 38]. Our analyses related to the net effect of smoking cessation after controlling for genetic and shared environmental family background also merit attention since genetics has proven to have a strong interaction between smoking status and BMI [10, 11, 20, 21]. In general, our within-pair results are in concordance with the series of MR meta-analyses testing the molecular mechanisms and causality behind smoking and BMI [10, 11, 20]. Importantly, twin analyses together with MR studies, both taking into account genetics and familial confounding behind the associations, have provided mutually supporting evidence about the causal nature of associations between smoking and BMI.

The study also has certain limitations. First, smoking and BMI were mainly self-reported values without information about the amount and duration of smoking, the time since smoking cessation, information about BMI prior to initiating/quitting smoking, information about other health behaviour factors such as alcohol consumption, energy supply and physical exercise. Unfortunately, not all included cohorts had these covariates available in a way that we could harmonize their use in this data. Furthermore, we are not aware of any smoking cessation studies in which pre-initiation weights would have been recorded, and this information may be subject to recall bias if reported later. There was no information regarding overall health status and the presence of non-communicable diseases (such as lung disease, heart disease and metabolic disease) among the participants. These diseases can confound BMI in any of the smoking behaviour groups. In the pairwise comparison, the twins in smoking discordant pairs have the same ethnic background same parental SES and also very similar own education [43, 44]. Thus, the effects of smoking exposure and disease are the remaining potential confounders. Given the relatively young age of the pairs (only 20% of the pairs aged 50 or more and 11% of the pairs aged 60 or more during any of the 10-year surveys), the effect of comorbidity in this data is likely to be small. Moreover, BMI is known to increase as adults age, at least until 60–70 years of age [30], and this increase is mainly due to an increase of fat deposits in mid-life [45, 46]. Even though current smoking is associated with lower BMI compared with never smoking, also current smokers tend to gain weight while ageing and this trend has been more evident in women [9, 35]. There is also evidence that longer and heavier exposure to smoking may increase especially accumulation of central adiposity and waist circumference [10]. Lack of information related to exposure of smoking in our data may have diluted the effect of smoking on BMI. However, our individual-based results regarding the association between smoking behaviours and BMI are in line with the previously reported WHO MONICA project [7]. Notably, even though BMI is shown to have strong correlation with body fat mass at the population level [47], we lack exact indicators for body adiposity in this data, as did a majority of related studies before. How smoking and changes in smoking behaviour are affecting BMI development, especially the development of adiposity in different body compartments (such as abdominal or subcutaneous), in different age groups requires further studies. In this study, the majority of the twin pairs were reporting their data close to each other within each 10-year period, but there were some twin pairs in which the reporting time gap between the co-twins within a 10-year period was a few years. However, there is evidence that long-term BMI discordance is rare in MZ pairs [48] and the effect of age difference within the pairs was controlled for in the co-twin analyses in this study. Notably, our analyses are also cross sectional in their nature. In this data 10 twin cohorts (50% of all included cohorts) included only one measurement point (one of the five 10-year periods) between 1960 and 2012. Finally, we did not stratify our analyses by geographical areas or birth cohorts in this study. Future studies analysing associations of smoking with BMI in different geographical or obesogenic environments and comparing associations in different birth cohorts are needed.

## Conclusion

Current smoking was associated with lower BMI and smoking cessation with higher BMI, independent of genetic and shared environmental familial factors. This association has not changed over time and was present in men and women. Tobacco smoking and quitting smoking do not appear to have substantial or permanent effects on the weight of adults, on average, since the BMI of persons who had initiated and then quit was about the same as that of their never smoking MZ co-twins. Even though smoking may reduce weight and smoking cessation may increase weight, smoking overall was not associated with a net weight increase as compared to never smokers. This information can alleviate concerns of weight gain in smokers who wish to quit smoking.

## Supporting information

S1 Table Sex-specific mean body mass index (BMI) values and standard deviations (SD) by smoking status, region, and the twin cohort (country) in the CODATwins database with 230,378 BMI and smoking observations. (DOCX)

S2 Table Individual-based and within-pair associations of current smoking with BMI compared with never smoking (reference) in twin individuals and in same-sex smoking discordant twin pairs (Twin1 = current / Twin2 = never) in the CODATwins database by sex, zygosity and time period. ^a^ Adjusted (age, age^2^ and twin cohort) linear regression coefficient with 95% confidence intervals. A robust variance estimator was used to adjust for the non-independence of observations within twin pairs.
^b^ A robust variance estimator was used to adjust for the non-independence of (repeated or paired) measurements during 1960–2012 in some twin individuals (or pairs).
^c^ Number of smoking discordant pairs (current vs never). Only one paired measurement was allowed for a 10-year period within a twin pair.
^d^ Number of smoking discordant pairs (current vs never) in within-pair measurements, 1960–2012.
^e^ Age-adjusted fixed-effect linear regression coefficient with 95% confidence intervals.
p-values: * 0.01≤ p <0.05, **0.001≤ p <0.01, *** p<0.001; statistically significant associations (i.e., regression coefficient) differs from zero, are in **bold**.
β = regression coefficient; BMI = body mass index; CI = confidence interval; DZ = dizygotic; m = number of within-pair measurements; MZ = monozygotic; n = number.
(DOCX)

S3 Table Individual-based and within-pair associations of former smoking with BMI compared with current smoking (reference) in twin individuals and in same-sex smoking discordant twin pairs (Twin1 = former / Twin2 = current) in the CODATwins database by sex, zygosity and time period. ^a^ Adjusted (age, age^2^ and twin cohort) linear regression coefficient with 95% confidence intervals. A robust variance estimator was used to adjust for the non-independence of observations within twin pairs.
^b^ A robust variance estimator was used to adjust for the non-independence of (repeated or paired) measurements during 1960–2012 in some twin individuals (or pairs).
^c^ Number of smoking discordant pairs (current vs former). Only one paired measurement was allowed for a 10-year period within a twin pair.
^d^ Number of smoking discordant pairs (current vs former) in within pair measurements, 1960–2012.
^e^ Age-adjusted fixed-effect linear regression coefficient with 95% confidence intervals.
p-values: * 0.01≤ p <0.05, **0.001≤ p <0.01, *** p<0.001; statistically significant associations (i.e., regression coefficient differs from zero) are in **bold**.
β = regression coefficient; BMI = body mass index; CI = confidence interval; DZ = dizygotic; m = number of within-pair measurements; MZ = monozygotic; n = number.
(DOCX)

S4 Table Individual-based and within-pair associations of former smoking with BMI compared with never smoking (reference) in twin individuals and in same-sex smoking discordant twin pairs (Twin1 = former / Twin2 = never) in the CODATwins database by sex, zygosity and time period. ^a^ Adjusted (age, age^2^ and twin cohort) linear regression coefficient with 95% confidence intervals. A robust variance estimator was used to adjust for the non-independence of observations within twin pairs.
^b^ A robust variance estimator was used to adjust for the non-independence of (repeated or paired) measurements during 1960–2012 in some twin individuals (or pairs).
^c^ Number of smoking discordant pairs (former vs never). Only one paired measurement was allowed for a 10-year period within a twin pair.
^d^ Number of smoking discordant pairs (former vs never) in within pair measurements, 1960–2012.
^e^ Age-adjusted fixed-effect linear regression coefficient with 95% confidence intervals.
p-values: * 0.01≤ p <0.05, **0.001≤ p <0.01, *** p<0.001; statistically significant associations (i.e., regression coefficient differs from zero) are in **bold.**
β = regression coefficient; BMI = body mass index; CI = confidence interval; DZ = dizygotic; m = number of within-pair measurements; MZ = monozygotic; n = number.
(DOCX)

S1 Text Contact information for the 21 twin cohorts. (DOCX)

## Abbreviations

95% CI: 95% confidence interval
β: Regression coefficient
BMI: Body mass index
DZ: Dizygotic (i.e., fraternal)
CODATwins: COllaborative project of Development of Anthropometrical measures in Twins
n: number of individuals or pairs
m: number of within-pair measurements
MZ: Monozygotic (i.e., genetically identical at the sequence level)
MR: Mendelian randomisation
SD: Standard deviation
