# Supplementary material for: Association of current and former smoking with body mass index: A study of smoking discordant twin pairs from 21 twin cohorts
Source: PLoS One. 2018 Jul 12;13(7):e0200140. doi: 10.1371/journal.pone.0200140 (PMC6042712; doi:10.1371/journal.pone.0200140)
Supplement: S1 Table — (DOCX) [file pone.0200140.s001.docx]

**S1 Table.** **Sex-specific mean body mass index (BMI) values and standard deviations (SD) by smoking status, region, and the twin cohort (country) in the CODATwins database with 230,378 BMI and smoking observations.**

| **Cohorts by regions**  **(country)** | **Number**  **of BMI observations**  **per twin cohort/ region**  **(% of males)** | **ALL (n=230,378)** | | | **MEN (n=118,733)** | | | **WOMEN (n=111,645)** | | |
| --- | --- | --- | --- | --- | --- | --- | --- | --- | --- | --- |
|  |  | **Smoking status** | | | **Smoking status** | | | **Smoking status** | | |
|  |  | **Never** | **Current** | **Former** | **Never** | **Current** | **Former** | **Never** | **Current** | **Former** |
|  |  | **Mean (SD)** | **Mean (SD)** | **Mean (SD)** | **Mean (SD)** | **Mean (SD)** | **Mean (SD)** | **Mean (SD)** | **Mean (SD)** | **Mean (SD)** |
| **Total** | **230,378 (52)** | **23.9 (3.7)** | **23.5 (3.5)** | **24.8 (3.7)** | **24.5 (3.2)** | **24.2 (3.2)** | **25.5 (3.3)** | **23.5 (4.0)** | **22.5 (3.7)** | **23.6 (4.0)** |
| **Europe** | **165,616 (48)** | **23.8 (3.6)** | **23.2 (3.4)** | **24.6 (3.6)** | **24.3 (3.1)** | **24.0 (3.2)** | **25.3 (3.3)** | **23.5 (3.8)** | **22.3 (3.5)** | **23.6 (3.8)** |
| Flanders (BEL) | 801 (48) | 22.1 (3.4) | 22.5 (3.6) | 22.4 (3.0) | 22.1 (2.9) | 22.6 (3.4) | 23.0 (2.4) | 22.1 (3.7) | 22.5 (3.9) | 22.0 (3.3) |
| FinnTwin12 (FIN) | 3,149 (44) | 23.1 (3.6) | 23.9 (4.0) | 23.8 (3.6) | 24.1 (3.2) | 24.5 (3.7) | 24.7 (3.5) | 22.4 (3.7) | 23.3 (4.2) | 23.1 (3.5) |
| FinnTwin16 (FIN) | 5,054 (46) | 22.9 (3.4) | 23.2 (3.5) | 23.5 (3.7) | 23.9 (3.2) | 24.0 (3.2) | 24.3 (3.6) | 22.2 (3.4) | 22.4 (3.6) | 22.8 (3.7) |
| FinnTwin Old (FIN) | 66,230 (48) | 24.0 (3.7) | 23.6 (3.5) | 24.9 (3.8) | 24.4 (3.2) | 24.3 (3.2) | 25.6 (3.4) | 23.8 (3.9) | 22.5 (3.6) | 23.7 (4.1) |
| Berlin (DEU) | 466 (35) | 23.4 (3.7) | 23.6 (4.0) | 25.2 (4.1) | 24.0 (3.7) | 24.1 (3.6) | 26.4 (3.5) | 23.1 (3.7) | 23.4 (4.2) | 24.2 (4.4) |
| Hungary (HUN) | 554 (30) | 24.4 (4.9) | 24.3 (4.4) | 24.9 (4.7) | 25.3 (4.4) | 24.7 (4.8) | 27.6 (4.8) | 24.0 (5.1) | 24.1 (4.3) | 23.6 (4.1) |
| Italy (ITA) | 4,978 (39) | 23.0 (3.7) | 23.0 (3.6) | 24.6 (3.9) | 24.4 (3.2) | 24.3 (3.1) | 25.9 (3.5) | 22.3 (3.7) | 22.1 (3.6) | 23.3 (3.8) |
| Norway (NOR) | 19,183 (47) | 23.0 (2.9) | 22.6 (2.9) | 23.6 (3.0) | 23.6 (2.5) | 23.6 (2.6) | 24.4 (2.6) | 22.6 (3.1) | 21.7 (2.8) | 22.5 (3.1) |
| Murcia (ESP) | 2,216 (43) | 27.2 (4.3) | 25.9 (4.1) | 27.6 (4.4) | 27.5 (3.9) | 27.1 (3.8) | 28.4 (4.0) | 27.1 (4.5) | 24.9 (4.1) | 26.0 (4.9) |
| SYMTS-adults (SWE) | 3,651 (100) | 23.3 (2.8) | 23.3 (2.8) | 23.6 (2.9) | 23.3 (2.8) | 23.3 (2.8) | 23.6 (2.9) | - | - | - |
| Swedish Twin (SWE) | 58,869 (47) | 23.9 (3.5) | 22.9 (3.4) | 24.6 (3.5) | 24.5 (3.1) | 23.7 (3.2) | 25.2 (3.2) | 23.5 (3.7) | 22.2 (3.4) | 23.8 (3.7) |
| Turkey (TUR) | 465 (55) | 22.2 (3.3) | 23.6 (3.4) | 27.0 (5.3) | 23.6 (2.9) | 24.0 (3.2) | 26.7 (4.9) | 21.2 (3.3) | 22.1 (3.7) | 27.5 (6.1) |
| **North America/ Australia** | **61,973 (61)** | **24.4 (4.0)** | **24.2 (3.7)** | **25.2 (3.8)** | **25.1 (3.3)** | **24.7 (3.3)** | **25.8 (3.4)** | **23.7 (4.4)** | **23.2 (4.4)** | **23.9 (4.4)** |
| Australia (AUS) | 2,357 (22) | 25.0 (4.7) | 25.8 (5.7) | 26.3 (4.8) | 25.9 (4.0) | 26.7 (4.7) | 27.2 (3.9) | 24.7 (4.9) | 25.5 (5.9) | 26.0 (5.1) |
| Queensland (AUT) | 24,826 (40) | 23.9 (4.0) | 23.8 (4.0) | 24.3 (4.0) | 24.4 (3.4) | 24.6 (3.5) | 25.3 (3.5) | 23.6 (4.3) | 23.1 (4.3) | 23.5 (4.2) |
| WWII (USA) | 21,959 (100) | 25.4 (2.9) | 24.6 (3.0) | 25.7 (3.0) | 25.4 (2.9) | 24.6 (3.0) | 25.7 (3.0) | - | - | - |
| VETSA (USA) | 1,225 (100) | 28.9 (4.4) | 27.9 (4.5) | 30.3 (4.8) | 28.9 (4.4) | 27.9 (4.5) | 30.3 (4.8) | - | - | - |
| Colorado (USA) | 1,819 (45) | 22.9 (3.9) | 23.3 (4.6) | 23.2 (4.1) | 23.6 (3.5) | 22.9 (3.9) | 23.4 (3.8) | 22.3 (4.1) | 23.5 (5.0) | 23.0 (4.3) |
| Mid-Atlantic (USA) | 9,787 (36) | 24.1 (4.2) | 23.8 (4.1) | 24.9 (4.1) | 24.7 (3.3) | 24.7 (3.5) | 25.9 (3.4) | 23.8 (4.4) | 23.2 (4.3) | 24.1 (4.4) |
| **East Asia** | **2,789 (39)** | **22.9 (3.2)** | **23.5 (3.1)** | **24.1 (3.1)** | **24.2 (3.0)** | **24.0 (3.0)** | **24.7 (2.9)** | **22.6 (3.2)** | **22.0 (3.1)** | **21.3 (2.4)** |
| Korea Twin-Fam (KOR) | 1,340 (38) | 22.6 (2.9) | 23.7 (3.3) | 24.3 (2.8) | 24.1 (2.7) | 24.2 (3.0) | 24.8 (2.7) | 22.4 (2.9) | 21.9 (3.7) | 21.9 (2.0) |
| Osaka (JPN) | 451 (26) | 20.9 (3.0) | 22.2 (2.8) | 22.6 (3.2) | 23.3 (3.7) | 22.8 (3.6) | 23.8 (2.8) | 20.4 (2.5) | 22.0 (2.6) | 20.6 (2.8) |
| Qingdao (CHN) | 998 (47) | 24.0 (3.2) | 23.9 (2.9) | 25.2 (3.3) | 24.6 (3.0) | 23.9 (2.9) | 25.2 (3.3) | 23.8 (3.3) | 22.3 (4.4) | 0 (0) |
