# Supplementary material for: Association of current and former smoking with body mass index: A study of smoking discordant twin pairs from 21 twin cohorts
Source: PLoS One. 2018 Jul 12;13(7):e0200140. doi: 10.1371/journal.pone.0200140 (PMC6042712; doi:10.1371/journal.pone.0200140)
Supplement: S2 Table — a Adjusted (age, age2 and twin cohort) linear regression coefficient with 95% confidence intervals. A robust variance estimator was used to adjust for the non-independence of observations within twin pairs. b A robust variance estimator was used to adjust for the non-independence of (repeated or paired) measurements during 1960–2012 in some twin individuals (or pairs). c Number of smoking discordant pairs (current vs never). Only one paired measurement was allowed for a 10-year period within a twin pair. d Number of smoking discordant pairs (current vs never) in within-pair measurements, 1960–2012. e Age-adjusted fixed-effect linear regression coefficient with 95% confidence intervals. p-values: * 0.01≤ p <0.05, **0.001≤ p <0.01, *** p<0.001; statistically significant associations (i.e., regression coefficient) differs from zero, are in bold. β = regression coefficient; BMI = body mass index; CI = confidence interval; DZ = dizygotic; m = number of within-pair measurements; MZ = monozygotic; n = number. (DOCX) [file pone.0200140.s002.docx]

**S2 Table**. **Individual-based and within-pair associations of current smoking with BMI compared with never smoking (reference) in twin individuals and in same-sex smoking discordant twin pairs (Twin1=current / Twin2=never) in the CODATwins database by sex, zygosity and time period.**

| **Time period** | **Individual-based** | **Within-pair** |  |  |  |
| --- | --- | --- | --- | --- | --- |
|  | **All twins as individuals** | **DZ pairs** |  | **MZ pairs** |  |
|  | **β (95% CI) ^a, b^** | **n ^c^ / m ^d^** | **β (95% CI) ^e^** | **n ^c^ / m ^d^** | **β (95% CI) ^e^** |
| **Men** (n=80,384) |  |  |  |  |  |
| 1960-69 | **-0.69 (-0.82, -0.55) ***** | 330 ^c^ | **-0.80 (-1.09, -0.51)** *** | 194 ^c^ | **-0.90 (-1.14, -0.65)** *** |
| 1970-79 | **-0.12 (-0.19, -0.04) **** | 1,261 ^c^ | **-0.23 (-0.38, -0.09)** ** | 402 ^c^ | **-0.44 (-0.59, -0.29)** *** |
| 1980-89 | **-0.12 (-0.20, -0.03) **** | 834 ^c^ | **-0.25 (-0.43, -0.06)** ** | 296 ^c^ | **-0.77 (-0.96, -0.58)** *** |
| 1990-99 | -0.03 (-0.13, 0.08) | 473 ^c^ | **-0.35 (-0.60, -0.09)** ** | 245 ^c^ | **-0.61 (-0.84, -0.37)** *** |
| 2000-12 | **-0.19 (-0.30, -0.08) **** | 601 ^c^ | -0.05 (-0.32, 0.21) | 398 ^c^ | **-0.71 (-0.92, -0.51)** *** |
| 1960-2012 ^b^ | **-0.19 (-0.25, -0.14) ***** | 3,499 ^d^ | **-0.24 (-0.35, -0.14)** *** | 1,535 ^d^ | **-0.57 (-0.70, -0.47)** *** |
|  |  |  |  |  |  |
| **Women** (n=76,210) |  |  |  |  |  |
| 1960-69 | no data |  | no data |  | no data |
| 1970-79 | **-0.40 (-0.49, -0.32) ***** | 1,401 | **-0.27 (-0.43, -0.12)** ** | 492 | **-0.71 (-0.87, -0.54)** *** |
| 1980-89 | **-0.42 (-0.53, -0.32) ***** | 892 | **-0.23 (-0.45, -0.01)** * | 455 | **-0.92 (-1.12, -0.73)** *** |
| 1990-99 | **-0.50 (-0.62, -0.37) ***** | 496 | **-0.99 (-1.30, -0.68)** *** | 261 | **-1.08 (-1.36, -0.80)** *** |
| 2000-12 | -0.09 (-0.21, 0.04) | 665 | -0.07 (-0.35, 0.22) | 432 | **-0.52 (-0.74, -0.30)** *** |
| 1960-2012 ^b^ | **-0.35 (-0.41, -0.28) ***** | 3,454 ^d^ | **-0.32 (-0.44, -0.19)** *** | 1,640 ^d^ | **-0.65 (-0.79, -0.52)** *** |

^a^ Adjusted (age, age^2^ and twin cohort) linear regression coefficient with 95% confidence intervals. A robust variance estimator was used to adjust for the non-independence of observations within twin pairs.

^b^ A robust variance estimator was used to adjust for the non-independence of (repeated or paired) measurements during 1960-2012 in some twin individuals (or pairs).

^c^ Number of smoking discordant pairs (current vs never). Only one paired measurement was allowed for a 10-year period within a twin pair.

^d^ Number of smoking discordant pairs (current vs never) in within-pair measurements, 1960-2012.

^e^ Age-adjusted fixed-effect linear regression coefficient with 95% confidence intervals.

p-values: * 0.01≤ p <0.05, **0.001≤ p <0.01, *** p<0.001; statistically significant associations (i.e., regression coefficient) differs from zero, are in **bold**.

β=regression coefficient; BMI=body mass index; CI=confidence interval; DZ=dizygotic; m=number of within-pair measurements; MZ=monozygotic; n=number
