# Supplementary material for: Association of current and former smoking with body mass index: A study of smoking discordant twin pairs from 21 twin cohorts
Source: PLoS One. 2018 Jul 12;13(7):e0200140. doi: 10.1371/journal.pone.0200140 (PMC6042712; doi:10.1371/journal.pone.0200140)
Supplement: S3 Table — a Adjusted (age, age2 and twin cohort) linear regression coefficient with 95% confidence intervals. A robust variance estimator was used to adjust for the non-independence of observations within twin pairs. b A robust variance estimator was used to adjust for the non-independence of (repeated or paired) measurements during 1960–2012 in some twin individuals (or pairs). c Number of smoking discordant pairs (current vs former). Only one paired measurement was allowed for a 10-year period within a twin pair. d Number of smoking discordant pairs (current vs former) in within pair measurements, 1960–2012. e Age-adjusted fixed-effect linear regression coefficient with 95% confidence intervals. p-values: * 0.01≤ p <0.05, **0.001≤ p <0.01, *** p<0.001; statistically significant associations (i.e., regression coefficient differs from zero) are in bold. β = regression coefficient; BMI = body mass index; CI = confidence interval; DZ = dizygotic; m = number of within-pair measurements; MZ = monozygotic; n = number. (DOCX) [file pone.0200140.s003.docx]

**S3 Table. Individual-based and within-pair associations of former smoking with BMI compared with current smoking (reference) in twin individuals and in same-sex smoking discordant twin pairs (Twin1=former / Twin2=current) in the CODATwins database by sex, zygosity and time period.**

| **Time period** | **Individual-based** | **Within-pair** |  |  |  |
| --- | --- | --- | --- | --- | --- |
|  | **All twins as individuals** | **DZ pairs** |  | **MZ pairs** |  |
|  | **β (95% CI) ^a, b^** | **n ^c^ / m ^d^** | **β (95% CI) ^e^** | **n ^c^ / m ^d^** | **β (95% CI) ^e^** |
| **Men** (n=80,384) |  |  |  |  |  |
| 1960-69 | **0.65 (0.52, 0.78) ***** | 511 ^c^ | **0.73 (0.48, 0.98)** *** | 369 ^c^ | **0.70 (0.62, 0.78)** *** |
| 1970-79 | **0.39 (0.31, 0.48) ***** | 1,301 ^c^ | **0.45 (0.31, 0.59)** *** | 593 ^c^ | **0.80 (0.60, 0.99)** *** |
| 1980-89 | **0.65 (0.56, 0.74) ***** | 1,081 ^c^ | **0.57 (0.40, 0.74)** *** | 673 ^c^ | **0.46 (0.32, 0.60)** *** |
| 1990-99 | **0.68 (0.56, 0.79) ***** | 522 ^c^ | **0.81 (0.56, 1.06)** *** | 291 ^c^ | **0.88 (0.73, 1.04)** *** |
| 2000-12 | **0.89 (0.77, 1.02) ***** | 574 ^c^ | **0.78 (0.51, 1.05)** *** | 468 ^c^ | **0.92 (0.70, 1.15)** *** |
| 1960-2012 ^b^ | **0.66 (0.61, 0.72) ***** | 3,989 ^d^ | **0.60 (0.51, 0.69) ***** | 2,394 ^d^ | **0.82 (0.62, 1.01)** *** |
|  |  |  |  |  |  |
| **Women** (n=76,210) |  |  |  |  |  |
| 1960-69 | no data |  | no data |  | no data |
| 1970-79 | **0.35 (0.23, 0.46) ***** | 717 ^c^ | **0.57 (0.38, 0.76)** *** | 381 ^c^ | **0.53 (0.36, 0.70)** *** |
| 1980-89 | **0.24 (0.11, 0.36) ***** | 686 ^c^ | **0.52 (0.29, 0.75)** *** | 584 ^c^ | **0.69 (0.52, 0.87)** *** |
| 1990-99 | **0.56 (0.41, 0.70) ***** | 451 ^c^ | **1.04 (0.72, 1.35)** *** | 355 ^c^ | **0.97 (0.71, 1.23)** *** |
| 2000-12 | **0.39 (0.25, 0.53) ***** | 543 ^c^ | **0.61 (0.31, 0.91)** *** | 450 ^c^ | **0.75 (0.53, 0.96)** *** |
| 1960-2012 ^b^ | **0.43 (0.36, 0.50) ***** | 2,397 ^d^ | **0.59 (0.47, 0.70) ***** | 1,770 ^d^ | **0.62 (0.51, 0.73) ***** |

^a^ Adjusted (age, age^2^ and twin cohort) linear regression coefficient with 95% confidence intervals. A robust variance estimator was used to adjust for the non-independence of observations within twin pairs.

^b^ A robust variance estimator was used to adjust for the non-independence of (repeated or paired) measurements during 1960-2012 in some twin individuals (or pairs).

^c^ Number of smoking discordant pairs (current vs former). Only one paired measurement was allowed for a 10-year period within a twin pair.

^d^ Number of smoking discordant pairs (current vs former) in within pair measurements, 1960-2012.

^e^ Age-adjusted fixed-effect linear regression coefficient with 95% confidence intervals.

p-values: * 0.01≤ p <0.05, **0.001≤ p <0.01, *** p<0.001; statistically significant associations (i.e., regression coefficient differs from zero) are in **bold**.

β=regression coefficient; BMI=body mass index; CI=confidence interval; DZ=dizygotic; m=number of within-pair measurements; MZ=monozygotic; n=number
