# Supplementary material for: Association of current and former smoking with body mass index: A study of smoking discordant twin pairs from 21 twin cohorts
Source: PLoS One. 2018 Jul 12;13(7):e0200140. doi: 10.1371/journal.pone.0200140 (PMC6042712; doi:10.1371/journal.pone.0200140)
Supplement: S4 Table — a Adjusted (age, age2 and twin cohort) linear regression coefficient with 95% confidence intervals. A robust variance estimator was used to adjust for the non-independence of observations within twin pairs. b A robust variance estimator was used to adjust for the non-independence of (repeated or paired) measurements during 1960–2012 in some twin individuals (or pairs). c Number of smoking discordant pairs (former vs never). Only one paired measurement was allowed for a 10-year period within a twin pair. d Number of smoking discordant pairs (former vs never) in within pair measurements, 1960–2012. e Age-adjusted fixed-effect linear regression coefficient with 95% confidence intervals. p-values: * 0.01≤ p <0.05, **0.001≤ p <0.01, *** p<0.001; statistically significant associations (i.e., regression coefficient differs from zero) are in bold. β = regression coefficient; BMI = body mass index; CI = confidence interval; DZ = dizygotic; m = number of within-pair measurements; MZ = monozygotic; n = number. (DOCX) [file pone.0200140.s004.docx]

**S4 Table. Individual-based and within-pair associations of former smoking with BMI compared with never smoking (reference) in twin individuals and in same-sex smoking discordant twin pairs (Twin1=former / Twin2=never) in the CODATwins database by sex, zygosity and time period.**

| **Time period** | **Individual-based** | **Within-pair** |  |  |  |
| --- | --- | --- | --- | --- | --- |
|  | **All twins as individuals** | **DZ pairs** |  | **MZ pairs** |  |
|  | **β (95% CI) ^a, b^** | **n ^c^ / m ^d^** | **β (95% CI) ^e^** | **n ^c^ / m ^d^** | **β (95% CI) ^e^** |
| **Men** (n=80,384) |  |  |  |  |  |
| 1960-69 | -0.03 (-0.18, 0.12) | 235 ^c^ | -0.07 (-0.38, 0.24) | 153 ^c^ | -0.10 (-0.35, 0.15) |
| 1970-79 | **0.28 (0.19, 0.37) ***** | 845 ^c^ | **0.22 (0.06, 0.37)** ** | 338 ^c^ | 0.02 (-0.14, 0.18) |
| 1980-89 | **0.54 (0.45, 0.62) ***** | 866 ^c^ | **0.32 (0.14, 0.51)** *** | 524 ^c^ | 0.11 (-0.06, 0.29) |
| 1990-99 | **0.65 (0.55, 0.75) ***** | 486 ^c^ | **0.46 (0.21, 0.72)** *** | 284 ^c^ | **0.31 (0.09, 0.54)** ** |
| 2000-12 | **0.70 (0.60, 0.81) ***** | 674 ^c^ | **0.73 (0.47, 0.98)** *** | 489 ^c^ | 0.11 (-0.09, 0.30) |
| 1960-2012 ^b^ | **0.47 (0.42, 0.53) ***** | 3,106 ^d^ | **0.36 (0.25, 0.46) ***** | 1,788 ^d^ | **0.13 (0.04, 0.23) ***** |
|  |  |  |  |  |  |
| **Women** (n=76,210) |  |  |  |  |  |
| 1960-69 | no data |  | no data |  | no data |
| 1970-79 | -0.06 (-0.17, 0.05) | 660 ^c^ | **0.29 (0.10, 0.49)** ** | 306 ^c^ | -0.18 (-0.36, 0.01) |
| 1980-89 | **-0.19 (-0.30, -0.07) **** | 726 ^c^ | **0.29 (0.06, 0.52)** * | 455 ^c^ | **-0.23 (-0.43, -0.04)** * |
| 1990-99 | 0.06 (-0.07, 0.19) | 600 ^c^ | 0.05 (-0.24, 0.34) | 352 ^c^ | -0.11 (-0.37, 0.15) |
| 2000-12 | **0.31 (0.19, 0.43) ***** | 760 ^c^ | **0.54 (0.26, 0.82)** *** | 583 ^c^ | **0.23 (0**.0**2, 0.43)** * |
| 1960-2012 ^b^ | **0.09 (0.01, 0.16) *** | 2,746 ^d^ | **0.27 (0.14, 0.40) ***** | 1,696 ^d^ | -0.04 (-0.16, 0.09) |

^a^ Adjusted (age, age^2^ and twin cohort) linear regression coefficient with 95% confidence intervals. A robust variance estimator was used to adjust for the non-independence of observations within twin pairs.

^b^ A robust variance estimator was used to adjust for the non-independence of (repeated or paired) measurements during 1960-2012 in some twin individuals (or pairs).

^c^ Number of smoking discordant pairs (former vs never). Only one paired measurement was allowed for a 10-year period within a twin pair.

^d^ Number of smoking discordant pairs (former vs never) in within pair measurements, 1960-2012.

^e^ Age-adjusted fixed-effect linear regression coefficient with 95% confidence intervals.

p-values: * 0.01≤ p <0.05, **0.001≤ p <0.01, *** p<0.001; statistically significant associations (i.e., regression coefficient differs from zero) are in **bold.**

β=regression coefficient; BMI=body mass index; CI=confidence interval; DZ=dizygotic; m=number of within-pair measurements; MZ=monozygotic; n=number
