# Supplementary material for: Association of current and former smoking with body mass index: A study of smoking discordant twin pairs from 21 twin cohorts
Source: PLoS One. 2018 Jul 12;13(7):e0200140. doi: 10.1371/journal.pone.0200140 (PMC6042712; doi:10.1371/journal.pone.0200140)
Supplement: S1 Text — (DOCX) [file pone.0200140.s005.docx]

| **S1 Text. Contact information for the 21 Twin Cohorts.** | | | |
| --- | --- | --- | --- |
| **Cohort name** | **Contact person** | **Institute** | **e-maill address/ Web-page** |
| East Flanders Twin Survey | Catherine Derom | Centre of Human Genetics, University Hospitals Leuven | c.derom@telenet.be |
| FinnTwin12 Study | Jaakko Kaprio | Department of Public Health, University of Helsinki, Finland | [jaakko.kaprio@helsinki.fi](mailto:jaakko.kaprio@helsinki.fi) |
| FinnTwin16 Study | Jaakko Kaprio | Department of Public Health, University of Helsinki, Finland | [jaakko.kaprio@helsinki.fi](mailto:jaakko.kaprio@helsinki.fi) |
| Older Finnish Twin Cohort Study | Jaakko Kaprio | Department of Public Health, University of Helsinki, Finland | [jaakko.kaprio@helsinki.fi](mailto:jaakko.kaprio@helsinki.fi) |
| Berlin Twin Registry HealthTwiSt | Andreas Busjahn | HealthTwiSt GmbH, Berlin, German | abusjahn@healthtwist.de |
| Hungarian Twin Registry | Adam Tarnoki | Department of Radiology, Semmelweis University, Budapest, Hungary | [tarnoki2@gmail.com](mailto:tarnoki2@gmail.com) |
| Italian Twin Registry | Maria Stazi | Istituto Superiore di Sanità - Centre for Behavioural Sciences and Mental Health, Rome, Italy | stazi@iss.it |
| Norwegian Twin Registry | Jennifer Harris | Norwegian Institute of Public Health, Department of Genes and Environment, Oslo, Norway | jennifer.harris@fhi.no |
| Murcia Twin Registry | Juan Ordoñana | Department of Human Anatomy and Psychobiology, University of Murcia, Murcia, Spain | ordonana@um.es |
| Swedish Young Men Twin Study | Patrik Magnusson | Department of Medical Epidemiology and Biostatistics, Karolinska Institutet, Stockholm, Sweden | patrik.magnusson@ki.se |
| Swedish Twin Registry | Patrik Magnusson | Department of Medical Epidemiology and Biostatistics, Karolinska Institutet, Stockholm, Sweden | [patrik.magnusson@ki.se](mailto:patrik.magnusson@ki.se) |
| Turkish Twin Cohort | Sevgi Öncel | Department of Statistics, Kırıkkale University, Kırıkkale, Turkey | syoncel@gmail.com |
| Twins Research Australia  (Australian Twin Registry) | John Hopper | Centre for Epidemiology and Biostatistics, The University of Melbourne, Melbourne, Australia | j.hopper@unimelb.edu.au |
| Queensland Twin Register | Nicholas Martin | QIMR Berghofer Medical Research Institute, Brisbane, Australia | nickm@qimr.edu.au |
| NAS-NRC Twin Registry |  | National Archive of Computerized Data on Aging (NACDA), USA | <https://doi.org/10.3886/ICPSR36234.v5> |
| Vietnam Era Twin Study of Aging | Carol Franz | University of California, San Diego, Department of Psychiatry, La Jolla, CA, USA | cfranz@ucsd.edu |
| Colorado Twin Registry | Robin Corley | Institute for Behavioral Genetics, Boulder, CO, USA | Robin.Corley@colorado.edu |
| Mid Atlantic Twin Registry | Hermine Maes | Department of Human and Molecular Genetics, Virginia Commonwealth University, Richmond, VA, USA | hermine.maes@vcuhealth.org |
| Korean Twin-Family Register | Joohon Sung | Department of Epidemiology, Seoul National University School of Public Health, Seoul, South Korea | jsung@snu.ac.kr |
| Osaka University Aged Twin Registry | Chika Honda | Osaka University Graduate School of Medicine, Osaka University, Japan | honda-ch@sahs.med.osaka-u.ac.jp |
| Qingdao Twin Cohort | Qihua Tan | Institute of Public Health, University of Southern Denmark, Odense, Denmark | qtan@health.sdu.dk |
